# Global trends and predictors of face mask usage during the COVID-19 pandemic

**DOI:** 10.1186/s12889-021-12175-9

**Published:** 2021-11-15

**Authors:** Elena Badillo-Goicoechea, Ting-Hsuan Chang, Esther Kim, Sarah LaRocca, Katherine Morris, Xiaoyi Deng, Samantha Chiu, Adrianne Bradford, Andres Garcia, Christoph Kern, Curtiss Cobb, Frauke Kreuter, Elizabeth A. Stuart

**Affiliations:** 1 Department of Mental Health, Johns Hopkins Bloomberg School of Public Health, 615 N. Wolfe St. W1033, Baltimore, MD 21205 USA; 2 Department of Biostatistics, Johns Hopkins Bloomberg School of Public Health, 615 N. Wolfe St. W1033, Baltimore, MD 21205 USA; 3 Facebook Research, Menlo Park, California USA; 4 Joint Program in Survey Methodology, University of Maryland College Park, College Park, MD USA; 5 University of Mannheim, Mannheim, Germany; 6 Department of Statistics, Ludwig Maximilians University, Munich, Germany; 7 Institute for Employment Research, Nuremberg, Germany

**Keywords:** COVID-19, SARS-CoV-2, Face mask, Mask usage

## Abstract

**Background:**

Guidelines and recommendations from public health authorities related to face masks have been essential in containing the COVID-19 pandemic. We assessed the prevalence and correlates of mask usage during the pandemic.

**Methods:**

We examined a total of 13,723,810 responses to a daily cross-sectional online survey in 38 countries of people who completed from April 23, 2020 to October 31, 2020 and reported having been in public at least once during the last 7 days. The outcome was individual face mask usage in public settings, and the predictors were country fixed effects, country-level mask policy stringency, calendar time, individual sociodemographic factors, and health prevention behaviors. Associations were modeled using survey-weighted multivariable logistic regression.

**Results:**

Mask-wearing varied over time and across the 38 countries. While some countries consistently showed high prevalence throughout, in other countries mask usage increased gradually, and a few other countries remained at low prevalence. Controlling for time and country fixed effects, sociodemographic factors (older age, female gender, education, urbanicity) and stricter mask-related policies were significantly associated with higher mask usage in public settings. Crucially, social behaviors considered risky in the context of the pandemic (going out to large events, restaurants, shopping centers, and socializing outside of the household) were associated with lower mask use.

**Conclusion:**

The decision to wear a face mask in public settings is significantly associated with sociodemographic factors, risky social behaviors, and mask policies. This has important implications for health prevention policies and messaging, including the potential need for more targeted policy and messaging design.

**Supplementary Information:**

The online version contains supplementary material available at 10.1186/s12889-021-12175-9.

## Background

In an effort to control and prevent the spread of the novel coronavirus disease 2019 (COVID-19), health organizations have recommended the use of a face covering or mask in public settings. Yet, despite growing evidence of the effectiveness of using face masks in reducing the transmission of COVID-19 [1–7], there is still a lack of knowledge regarding mask-wearing behaviors on a global scale. In particular, it is still unclear how mask-wearing behavior has changed over time, how trends have varied across countries throughout this pandemic, and whether individual or country-level factors are associated with mask-wearing. These questions are critical to better understand and target behaviors that are considered risky in the context of the pandemic, across different individuals and regions; clarify and fine-tune public health messaging around face mask usage during the pandemic, and, more generally, help better design prevention campaigns in future public health emergencies.

To this date, few studies have rigorously examined global trends and individual predictors of face mask usage during the COVID-19 pandemic, primarily simply documenting rates of mask usage [6–8]. Previous work has examined sociodemographic factors and individual beliefs and attitudes as predictors of mask-wearing during other health emergencies [9], such as SARS-Cov-1 and H1N1 [10–14]. In addition, most previous studies used small non-random samples (e.g., ~ 300–5000 self-selected participants), from which it is difficult to learn about mask usage on a general population scale. Previous studies also generally had a limited time frame (e.g., one or two months), and/or narrow geographical coverage (e.g., one or only a few countries). Further, most did not conduct statistical analyses that jointly examined individual-and country-level factors that may explain differences in mask-wearing behavior.

In general, better understanding the social and regional determinants of behavioral patterns is crucial to adequately adapt health policies and communication campaigns to diverse populations and make them more effective at reaching intended audiences considering their different contexts and needs [15, 16]. Taking this into account is of utmost importance for the design of public health responses in the context of the ongoing global pandemic, such as those related to face mask usage and lockdown measures, which ultimately address—in most cases— individual decisions that might be influenced by social, political, and economic environments.

The main objective of this study was, therefore, to examine the evolution of mask usage across different countries over time during the COVID-19 pandemic and assess whether individual and country-level factors were associated with the decision to wear a mask. For this, we leveraged a novel dataset from the University of Maryland Social Data Science Center COVID-19 Trends and Impact Survey (CTIS) [17], conducted in partnership with Facebook, which has tracked mask usage, sociodemographic characteristics, health indicators, and health prevention behaviors on a daily basis since April 2020. To the best of our knowledge, the CTIS is currently the largest data collection effort systematically monitoring mask usage and other social responses to the ongoing COVID-19 pandemic at a global scale with representativeness at a country-level. We used data from respondents from 38 countries who were randomly selected to take this survey between April 23, 2020 to October 31, 2020 and who reported having been in public at least once during the last 7 days, which yielded approximately 13 million adults. To this date, no other study has used data with such characteristics to formally examine trends and predictors of mask usage worldwide.

## Methods

### The global COVID-19 trends and impact survey

The CTIS is an ongoing repeated daily cross-sectional survey conducted by the University of Maryland (Global) and Carnegie Mellon University (US) in partnership with Facebook, Inc. It asks various questions related to symptoms, testing, preventive behaviors, mental health, and more. The UMD Global CTIS was launched on April 23, 2020 in > 200 countries and territories (Supplementary Table 1) and the US CTIS was launched on April 6, 2020. The survey instrument, sampling design, and weighting methodology are described in more detail below. The Symptom Survey was reviewed and approved by the Institutional Review Boards of both the University of Maryland and Carnegie Mellon University.

The survey instrument was developed by public health and survey experts [15], and included the following sections: COVID-19 related symptoms, testing, contact history, preventive behavior (e.g., face mask usage, hand washing, social distancing, etc.), mental health, economic security, and basic demographics. The questionnaire is publicly available online [18, 19], and is translated into 56 locales (listed in Supplementary Table 1).

The sampling methodology for the CTIS has been described previously [20]. Briefly, the sampling frame is composed of daily active Facebook users who are > = 18 years, living within 200+ countries or territories, and using one of the supported languages. This coverage ensures that > 95% of Facebook users are eligible. Every day, Facebook invites a stratified random sample to take the survey with an invitation at the top of their Facebook News Feed, with the sampling strata defined as the administrative boundaries within countries or territories [21]. Those who view the invitation and are interested in taking the survey are redirected to an off-Facebook survey administered by the academic partners. Facebook does not share or receive data from the academic partners other than a list of random identification numbers of those who completed the survey to calculate and share survey weights.

The details of the weighting methodology have been described previously [20]. Briefly, Facebook employs a two-stage weighting process to minimize bias related to non-response and coverage. In the first step, inverse propensity score weighting is used to adjust for non-response bias by making the sample more representative of the sampling frame of Facebook users. As stated above, Facebook only receives a list of identification numbers that indicate who completed the survey; therefore, the covariates used in this step are obtained from internal Facebook data, which consist of self-reported age, gender, geographical variables, and other attributes that have been found internally to correlate well with survey response [22]. At the second stage post-stratification or raking is used to equate the distribution of age and gender among the Facebook population to benchmarks from the United Nations Population Division 2019 World Population Projections, and first administrative level region benchmarks from publicly available population density maps [23].

### Study population

This analysis included adult participants who responded to the UMD Global CTIS from April 23rd, 2020 until October 31st, 2020. We did not include responses from the US Symptom Survey in this analysis, as the question on face mask usage was not incorporated into the US questionnaire until September 2020. Since some of the 200+ countries and territories have relatively small sample sizes, with high variability in responses, we focused on 38 countries based on the following criteria: countries that are considered either members, candidates, or key partners of the Organisation for Economic Co-operation and Development (OECD) convention [24], or countries with a sample size > 600,000 during our study period (Table 1). Over the course of field collection in the selected 38 countries, 741,496,298 Facebook users saw the survey invitation; 36,525,312 opened the survey invitation; and 18,730,575 responded to the survey. Of those, 1,020,188 reported being in public in the past 7 days. Missingness on the predictors ranged from 2 to 13% per variable, which overall resulted in 27% of the survey respondents being excluded, leading to a final analysis sample of 13,723,810.

**Table 1:** List of countries included in the analysis

Country	Total responses	Complete responses^a^	Analytic sample^b^
	18,730,575 (100%)	14,552,118 (77.69%)	13,723,810 (73.27%)
Argentina	659,009 (3.52%)	484,352 (2.59%)	435,134 (2.32%)
Australia	325,885 (1.74%)	266,113 (1.42%)	254,095 (1.36%)
Austria	138,777 (0.74%)	113,097 (0.6%)	111,133 (0.59%)
Belgium	142,999 (0.76%)	101,548 (0.54%)	97,550 (0.52%)
Brazil	2,322,508 (12.4%)	1,788,903 (9.55%)	1,700,210 (9.08%)
Bulgaria	96,459 (0.51%)	75,085 (0.4%)	72,191 (0.39%)
Canada	417,071 (2.23%)	346,718 (1.85%)	329,517 (1.76%)
Chile	324,447 (1.73%)	256,195 (1.37%)	229,449 (1.22%)
Colombia	574,169 (3.07%)	449,043 (2.4%)	394,540 (2.11%)
Costa Rica	167,986 (0.9%)	131,144 (0.7%)	115,907 (0.62%)
Czech Republic	213,108 (1.14%)	168,216 (0.9%)	162,533 (0.87%)
Denmark	306,917 (1.64%)	257,938 (1.38%)	254,406 (1.36%)
Finland	157,593 (0.84%)	133,380 (0.71%)	125,433 (0.67%)
France	708,994 (3.79%)	459,218 (2.54%)	442,412 (2.36%)
Germany	763,760 (4.08%)	628,053 (3.35%)	619,066 (3.31%)
Greece	197,813 (1.06%)	163,753 (0.87%)	154,268 (0.82%)
Hungary	320,668 (1.71%)	255,230 (1.36%)	242,901 (1.3%)
India	1,083,384 (5.78%)	728,852 (3.89%)	642,297 (3.43%)
Indonesia	547,797 (2.92%)	398,395 (2.13%)	370,107 (1.98%)
Ireland	163,006 (0.87%)	131,488 (0.7%)	125,645 (0.67%)
Israel	193,693 (1.03%)	156,551 (0.84%)	150,858 (0.81%)
Italy	989,919 (5.29%)	796,122 (4.25%)	775,840 (4.14%)
Japan	1,418,201 (7.57%)	1,178,538 (6.29%)	1,163,828 (6.21%)
Mexico	1,831,010 (9.78%)	1,425,019 (7.61%)	1,302,477 (6.95%)
Netherlands	355,421 (1.9%)	294,844 (1.57%)	265,162 (1.42%)
New Zealand	125,601 (0.67%)	101,695 (0.54%)	96,472 (0.52%)
Norway	205,460 (1.1%)	171,999 (0.92%)	157,285 (0.84%)
Poland	388,553 (2.07%)	265,143 (1.42%)	257,927 (1.38%)
Portugal	353,606 (1.89%)	247,065 (1.32%)	238,697 (1.27%)
Romania	385,949 (2.06%)	308,116 (1.64%)	293,426 (1.57%)
Russia	273,870 (1.46%)	214,795 (1.15%)	201,447 (1.08%)
Slovenia	43,665 (0.23%)	35,613 (0.19%)	34,606 (0.18%)
South Africa	235,188 (1.26%)	189,818 (1.01%)	179,337 (0.96%)
Spain	659,951 (3.52%)	518,427 (2.77%)	503,994 (2.69%)
Sweden	484,930 (2.59%)	409,192 (2.18%)	385,530 (2.06%)
Switzerland	148,638 (0.79%)	115,230 (0.62%)	112,087 (0.6%)
Turkey	480,588 (2.57%)	359,907 (1.92%)	336,594 (1.8%)
United Kingdom	523,982 (2.8%)	427,323 (2.28%)	389,449 (2.08%)

^a^Full sample for the primary model (April 23, 2020 - October 31, 2020)

^b^Complete cases (analytic sample) included in the primary model (April 23, 2020 - October 31, 2020)

### Outcome variable definition

Our outcome was face mask usage, based on the survey question: “In the last 7 days, how often did you wear a mask when in public?” The response options were “All of the time”, “Most of the time”, “Some of the time”, “A little of the time”, “None of the time”, or “I have not been in public during the past 7 days”. We defined face mask usage as a binary variable: 1 if the respondent reported wearing a mask all or most of the time, and 0 otherwise.

### Predictor measurement

We included several individual and country-level factors that could be associated with face mask usage based on a priori hypotheses and existing literature. Individual-level predictors included age, gender, standardized years of education, urbanicity (defined as living in a city versus town, village, or rural area), and the following reported social behaviors from the last 24 h: working outside the household, going to a market/grocery store/pharmacy, going to a restaurant/cafe/shopping center, spending time with someone outside their household, and attending a public event with more than 10 people. We also included whether the respondent reported ever being tested for COVID-19, and two variables capturing individual economic aspects: worried about household finances and worked in the last 7 days. The three variables on years of education, financial worry, and employment status in the last 7 days were added to the survey on June 27, 2020; therefore, data on these items were not available earlier than this date.

Country-level predictors were country fixed effects, the (time-varying) presence of official policies related to face masks, and the (time-varying) incidence of COVID-19 disease. The country-level mask usage policies were obtained from the University of Oxford Our World in Data’s COVID-19 dataset, which contains daily country-level policies on the use of face coverings outside-of-the-home. The policies are graded from 0 to 5 and reflect the strength of the policy (i.e., no policy, recommended, required in some specified places, required in all shared/public spaces, required at all times) for each country [25]. We generated standardized weekly averages of this mask-wearing policy stringency index for each country, and included the index as a continuous variable in the model. Country-day-level COVID-19 cases were obtained from the Johns Hopkins University Center for Systems Science and Engineering’s repository [26], which we used as a standardized seven-day lagged average to measure the association between the rate of COVID-19 cases during the last 7 days and the individual’s decision to wear a mask.

### Statistical analysis

In addition to examining descriptive statistics, a survey-weighted multivariable logistic regression model was used to formally assess whether individual and country-level factors were associated with mask-wearing. All statistical analyses were performed in R (version 4.0.3), using the R survey package (version 4.0) to account for the sampling design. We estimated two separate models to accommodate the fact that the three questions capturing socioeconomic factors (financial worry, years of education, and employment status in the last 7 days) were added later in field collection. The primary model included the entire sample from April 23, 2020, through October 31, 2020, with all predictors described above except for the three not available before June 27, 2020. A secondary model was fit with a narrower time period spanning from June 27, 2020 until October 31, 2020, to include the additional three socioeconomic factors. We included month as a categorical variable in all models.

## Results

### Evolution of mask usage over time

**Fig. 1:**
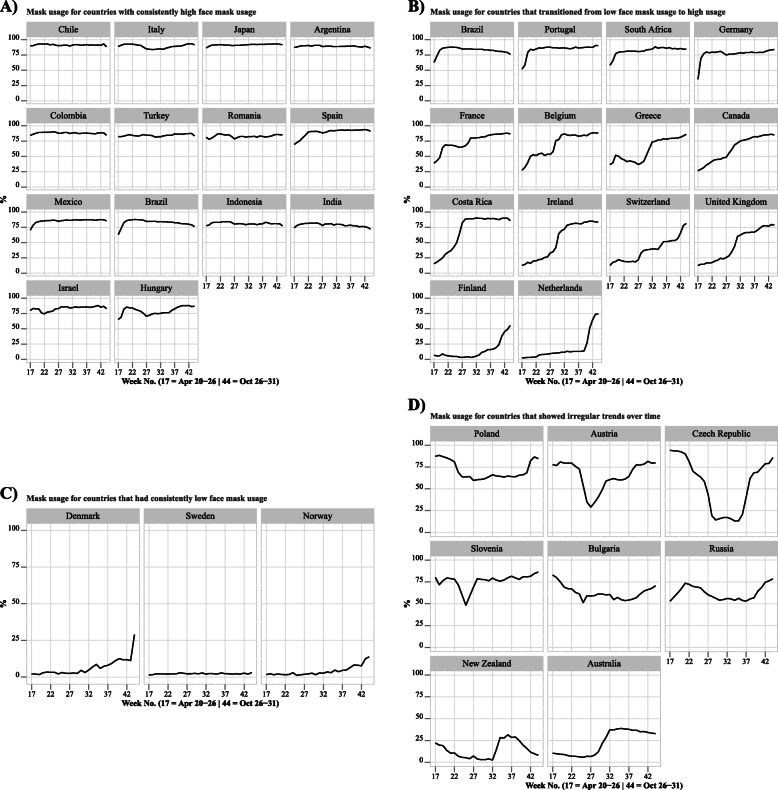
Weighted self-reported weekly mask usage prevalence by country, (Weights adjust each country sample to their corresponding national population.) grouped by A) countries with consistently high face mask usage, B) countries that transitioned from low to high face mask usage, C) countries that had consistently low face mask usage, D) countries that showed irregular trends over time. Panel 1A) Mask usage for countries with consistently high face mask usage. Panel 1B) Mask usage for countries that transitioned from low face mask usage to high usage. Panel 1C) Mask usage for countries that had consistently low face mask usage. Panel 1D) Mask usage for countries that showed irregular trends over time

Trends over time across the 38 countries (Fig. 1) suggested considerable heterogeneity in self-reported mask-wearing in public across countries. Some countries had consistently high mask usage (> 75%) from April until October (ex: Chile, Italy, Japan, Argentina, Colombia, Turkey, Romania, etc.) (Fig. 1A). In some other countries, mask usage was relatively low in April, but eventually increased and remained at higher levels (ex: Brazil, Portugal, South Africa, Germany, France, Belgium, Greece, Canada, etc.) (Fig. 1B). Mask usage was consistently low (< 25%) in certain countries (ex: Denmark, Sweden, and Norway) (Fig. 1C), and was more irregular in others (ex: Austria, Czech Republic, Slovenia, etc.) (Fig. 1D).

### Predictors of mask usage

Results from the logistic regression model confirmed the observed cross-country mask-wearing trends over time, with individuals from the vast majority of countries — particularly of Northern Europe — being significantly less likely to wear a mask when in public than individuals in Japan (the referent country). Individuals were more likely to wear a mask in later months (May: OR 1.71, 95% CI [1.69, 1.75]; June: OR 1.95, 95% CI [1.92, 1.99]; July: OR 2.01, 95% CI [1.97, 2.05]; August: OR 2.60, 95% CI [2.55, 2.65]; September: OR 2.74, 95% CI [2.69, 2.80]; October: OR 3.40, 95% CI [3.32, 3.48]) (Table 2).

**Table 2:** Weighted logistic regression results on the associations of various individual-level and country-level variables with face mask use^a^

	Primary model: From April 22 until October 31		Secondary model: From June 27 until October 31	
	Odds ratio (95% CI)	P	Odds ratio (95% CI)	P
**Time (month)**				
**April**	*ref*		Not available	
**May**	1.72 (1.69, 1.75)	< 0.001	Not available	
**June**	1.95 (1.92, 1.99)	< 0.001	*ref*	
**July**	2.01 (1.97, 2.05)	< 0.001	1.14 (1.11, 1.17)	< 0.001
**August**	2.60 (2.55, 2.65)	< 0.001	1.45 (1.42, 1.48)	< 0.001
**September**	2.74 (2.69, 2.80)	< 0.001	1.51 (1.48, 1.55)	< 0.001
**October**	3.40 (3.32, 3.48)	< 0.001	1.90 (1.85, 1.96)	< 0.001
**COVID-19 test ever taken**				
**No**	*ref*		*ref*	
**Yes**	1.59 (1.57, 1.61)	< 0.001	1.55 (1.53, 1.58)	< 0.001
**Age**				
**18–24 years**	*ref*		*ref*	
**25–34 years**	1.22 (1.20, 1.23)	< 0.001	1.16 (1.14, 1.18)	< 0.001
**35–44 years**	1.34 (1.32, 1.36)	< 0.001	1.23 (1.21, 1.26)	< 0.001
**45–54 years**	1.43 (1.41, 1.45)	< 0.001	1.32 (1.29, 1.34)	< 0.001
**55–64 years**	1.42 (1.40, 1.44)	< 0.001	1.28 (1.25, 1.30)	< 0.001
**65+ years**	1.47 (1.45, 1.50)	< 0.001	1.27 (1.24, 1.29)	< 0.001
**Gender**				
**Male/other**	*ref*		*ref*	
**Female**	1.70 (1.69, 1.71)	< 0.001	1.75 (1.73, 1.77)	< 0.001
**Living in an urban area**				
**No**	*ref*		*ref*	
**Yes**	1.40 (1.39, 1.41)	< 0.001	1.43 (1.41, 1.44)	< 0.001
**Gone out to work outside in the last 24 h**				
**No**	*ref*		*ref*	
**Yes**	0.98 (0.98, 0.99)	< 0.001	0.98 (0.97, 0.99)	< 0.001
**Gone out to a market, grocery store or pharmacy in the last 24 h**				
**No**	*ref*		*ref*	
**Yes**	1.07 (1.06, 1.08)	< 0.001	1.01 (1.09, 1.11)	< 0.001
**Gone out to a restaurant, café, or shopping center in the last 24 h**				
**No**	*ref*		*ref*	
**Yes**	0.77 (0.77, 0.78)	< 0.001	0.76 (0.75, 0.77)	< 0.001
**Spent time with someone outside their household in the last 24 h**				
**No**	*ref*		*ref*	
**Yes**	0.72 (0.72, 0.73)	< 0.001	0.70 (0.69, 0.71)	< 0.001
**Attended a public event with more than 10 people in the last 24 h**				
**No**	*ref*		*ref*	
**Yes**	0.45 (0.44, 0.45)	< 0.001	0.46 (0.46, 0.47)	< 0.001
**Mask policy stringency score**				
**Per 1 standard deviation**	1.58 (1.58, 1.59)	< 0.001	1.50 (1.48, 1.51)	< 0.001
**Seven day lagged COVID-19 cases**				
**Per 1 standard deviation**	0.93 (0.93, 0.94)	< 0.001	0.98 (0.98, 0.99)	< 0.001
**Worked in the last 7 days**				
**No**	Not available		*ref*	
**Yes**	Not available		0.98 (0.97, 0.99)	< 0.001
**Worried about household finances**				
**No**	Not available		*ref*	
**Yes**	Not available		0.88 (0.87, 0.89)	< 0.001
**Years of education**				
**Per 1 year**	Not available		1.07 (1.07, 1.08)	< 0.001

^a^Dependent variable: mask usage (binary), type: Analysis of complex survey design; link function: logit. Model includes fixed effects by country (not shown in Table). Pseudo *R*^2^ = 0.27 for the primary model; Pseudo *R*^2^ = 0.26 for secondary model.

Demographic, behavioral, and policy-related factors were significantly associated with wearing a face mask in public, even after controlling for time and country fixed effects. Of the demographic factors, female gender (OR 1.70, 95% CI [1.69, 1.71]), living in an urban area (OR 1.40, 95% CI [1.39, 1.41]), and older age (age 25–34: OR 1.22, 95% CI [1.20, 1.23]; age 35–44: OR 1.34, 95% CI [1.32, 1.36]; age 45–54: OR 1.43, 95% CI [1.41, 1.45]; age 55–64: OR 1.42, 95% CI [1.40, 1.44]); age 65+: OR 1.47, 95% CI [1.45, 1.49]) were positively associated with wearing a face mask.

Of the behavioral factors, going to a market, grocery store, or pharmacy was associated with higher mask use (OR 1.07, 95% CI [1.06, 1.08]), whereas more optional or risky behaviors [22] were associated with lower mask use. More specifically, behaviors associated with lower mask use were attending large public events (OR 0.45, 95% CI [0.44, 0.45]), socializing outside of the home [OR 0.72, 95% CI [0.72, 0.73]), and going to a restaurant, cafe, or shopping center (OR 0.77, 95% CI [0.77, 0.78]). Other significant behavioral factors examined were working outside from home, which was associated with lower mask usage (OR 0.98, 95% CI [0.97, 0.99]), and having been tested for COVID-19, which was associated with higher mask use (OR 1.59, 95% CI [1.57, 1.61]).

Regarding country-level factors, we observed that more strict policies were associated with higher mask usage (OR 1.58, 95% CI [1.57, 1.59]), while lagged COVID-19 cases were (OR 0.93, 95% CI [0.92, 0.94]) associated with less mask-wearing.

In the secondary model, which included data from late-June onwards and the three additional socioeconomic variables (financial worry, years of education, and employment status in the last 7 days), the aforementioned demographic, behavioral, and policy-related factors remained significantly associated with face mask usage. The three additional socioeconomic variables were significantly associated with mask-wearing: higher years of education was associated with higher use (OR 1.07, 95% CI [1.07, 1.08]) while financial worry and working in the last 7 days were associated with lower use (financial worry: OR 0.88, 95% CI [0.87, 0.89]; being employed in the last 7 days: OR 0.98, 95% CI [0.97, 0.99]) (Table 2).

**Fig. 2:**
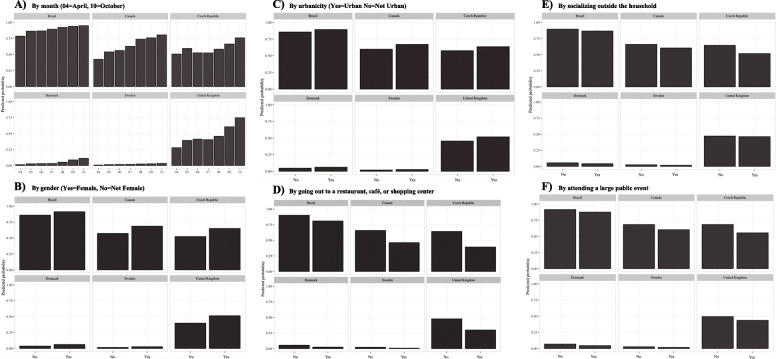
Predicted probability of face mask usage by individual characteristics for selected countries given various categories of A) month, B) gender, C) urbanicity, D) having gone to a restaurant, café or shopping center, E) having socialized outside of the household, and D) having attended a large public event. Panel 2A) By month (04 = April, 10 = October). Panel 2B) By gender (Yes = Female, No = Not Female). Panel 2C) By urbanicity (Yes = Urban, No = Not Urban). Panel 2D) By going out to a restaurant, café, or shopping center. Panel 2E) By socializing outside the household. Panel 2F) By attending a large public event

Figure 2 depicts the predicted probabilities of wearing a face mask for a few covariates. The results demonstrate that overall, the probabilities of mask-wearing increased over time from April until November but the extent to which the probabilities increased over time varied considerably depending on country (ranging from ~ 1% increase in Sweden to 50% increase in the United Kingdom; Fig. 2A). The probability of mask-wearing was also higher among individuals who identify as females (Fig. 2B) or are living in cities (Fig. 2C), while it was lower among those who have gone out to a restaurant/shopping center (Fig. 2D), socialized outside of the household (Fig. 2E), or attended a large public event (Fig. 2F). The probabilities varied depending on the country.

## Discussion

In this multi-national sample of over 13 million adults from 38 countries, we found considerable heterogeneity in mask use across countries throughout the COVID-19 pandemic, and some cross-country differences were statistically significant even after adjusting for individual- and country-level factors, such as time-varying mask-wearing policy stringency. More specifically, in 13 countries —most of them Latin American or Asian— mask usage prevalence stayed at 70% or higher throughout our study period, whereas in Denmark, Sweden, and Norway, mask usage has consistently remained below 15%. In most other countries, mask usage was low in April and gradually reached higher levels, although the pace at which this happened varied widely across these countries. A few other countries showed irregular trends over time. See Tables 1 and 3 for more sample characteristics.

**Table 3:** Weighted distribution of respondent characteristics among 13,723,810 respondents who reported being in public in the past 7 days and provided complete responses

	Overall (Unweighted *N* = 13,723,810)			Mask usage = 1^a^ (Unweighted *N* = 10,610,836)			Mask usage = 0^b^ (Unweighted *N* = 3,112,974)		
Sex	%			%			%		
Female	43.15			43.96			39.03		
Male	56.64			55.85			60.66		
Other	0.20			0.18			0.30		
Age	%			%			%		
18–34	14.68			14.71			14.52		
25–34	26.29			26.97			22.83		
35–44	18.76			18.84			18.34		
45–54	18.3			18.24			18.61		
55–64	10.70			10.40			12.23		
> = 65	11.26			10.84			13.44		
Current location	%			%			%		
Urban	46.37			44.33			56.75		
Non-urban	53.63			55.67			43.25		
Gone to work outside in the last 24 h	%			%			%		
Yes	36.23			35.63			39.29		
No	63.76			64.36			60.71		
Gone to a market, grocery store, or pharmacy in the last 24 h	%			%			%		
Yes	65.79			65.24			68.58		
No	35.96			34.76			31.42		
Gone to a restaurant, café, or shopping center in the last 24 h	%			%			%		
Yes	25.36			23.84			33.11		
No	74.64			76.15			66.89		
Spent time with a non-same household member in the last 24 h	%			%			%		
Yes	43.16			40.70			55.68		
No	56.84			59.29			44.32		
Attended a public event with more than 10 people in the last 24 h	%			%			%		
Yes	10.31			9.65			17.62		
No	89.05			90.35			82.38		
Tested for COVID-19	%			%			%		
Yes	12.94			13.36			10.76		
No	87.06			86.64			89.22		
Worried about household finances in the next month^c^	%			%			%		
Yes	21.01			21.35			17.68		
No	78.99			78.65			82.32		
Worked for pay in the last 7 days^c^	%			%			%		
Yes	53.10			53.84			58.79		
No	46.90			46.16			41.21		
Years of education^c^	Q1	Q2	Q3	Q1	Q2	Q3	Q1	Q2	Q3
	10	14	17	11	15	17	9	13	16

^a^Primary model (full analytic sample; April 23, 2020 - October 31, 2020)

^b^Secondary model (June 27, 2020 - October 31, 2020)

^c^Variable only included in the secondary model, fit with the narrower time period in which it was available

These differences suggest that there may be unobserved underlying institutional or cultural phenomena across countries that contribute to the adoption of mask-wearing. Consequently, pre-existing social norms and experiences related to mask-wearing within countries should be taken into consideration when shaping or analyzing mask-related policy guidelines. It is challenging to compare and contrast our mask use estimates or trends to those from other data sources, since this is, to the best of our knowledge, the first study that describes global longitudinal trends of face mask-wearing during the COVID-19 pandemic using samples of this scale. However, we found that the trends of mask use for some countries in our study (e.g., France, Germany, United Kingdom, and Sweden) are broadly similar to those reported by other online survey platforms such as YouGov’s COVID-19 Public Monitor [27].

Our findings also show that certain demographic factors; namely*,* older age, female gender, urbanicity, and higher education levels are associated with mask use. This is in line with previous studies reporting that age, gender, and education are significant predictors of face mask usage in the context of past outbreaks, such as SARS-Cov-1 and H1N1 [10–14]. Moreover, while it is not in the scope of this study to investigate the mechanisms through which age and gender affect mask usage, it has been documented that, in part due to peer effects and social role models, both female and older individuals tend to engage more in health-preventive behaviors, relative to male and younger individuals [11, 28, 29].

Less previous work has focused on urbanicity and mask wearing. One study conducted in Australia reported that those living in rural areas as opposed to urban areas are more likely to wear a face mask [11]. In contrast, we observed that those living in urban areas are more likely to wear a mask. Notably, the previous study was conducted in the context of an anticipated outbreak scenario, not during an actual global pandemic, and was focused in just one country.

Notably, we found that, even controlling for sociodemographic and country-level factors, social behaviors were differentially associated with wearing a mask. Specifically, social behaviors deemed more optional and risky in the context of the current pandemic, [30] such as going out to large public events, restaurants, cafes, shopping centers, or socializing outside of the household were associated with lower face mask usage. Other behaviors that also take place outdoors but may be less optional, such as going to a market, grocery store, or pharmacy, were associated with higher mask use. These results suggest that those who voluntarily engage in risky social activities during the pandemic are also less likely to wear a mask, which highlights a critical target for public health intervention, as these groups may contribute to higher risks of COVID-19 spread [31]. Finally, our study found that, even controlling for COVID-19 cases, time and country-fixed effects, stricter country-level policies around mask-wearing were associated with higher mask use. Taken together, these two results suggest that more emphasis should be made on designing targeted mask wearing policies in settings where individuals are less likely to do so.

This study has some limitations. First, years of education, financial worry, and employment status were not collected throughout the full field collection period, even though these may be important covariates to examine in association with mask use. To address this, however, we fit a secondary model that did include these three variables using the narrower time period during which they were collected, and found that the results for most associations remained very similar. Second, given our non-experimental study design, we cannot infer any causation from our findings.

Despite these limitations, there are several strengths to this study. Our analysis leveraged the largest ongoing, data collection related to COVID-19 symptoms and behaviors, which allowed us to examine and compare face mask usage trends across many countries and include a long time period spanning 7 months. We also simultaneously examined individual and country-level characteristics in our models, which allowed us to more adequately model the individual decision to wear a mask. Lastly, while the Facebook user base varies in its composition by country, we do not expect the trends to be affected, for there are no reported large shifts in the Facebook user base within countries during the study period.

In sum, our findings provide a better understanding of who are more or less likely to wear face masks in public during an outbreak and suggest that public health messaging should better target individuals who do not wear face masks in public as frequently. This has important implications for health prevention policies and messaging in the context of the ongoing and future public health emergencies, as they highlight important differences in mask usage between countries, as well as the importance of better targeting specific subpopulations when designing such policies and messaging campaigns.

## Conclusions

This study is the first to examine mask-wearing throughout the COVID-19 pandemic worldwide. In summary, it shows that various sociodemographic factors, such as older age, female gender, higher education, and urbanicity, are associated with higher face mask usage, while more risky social behaviors, such as going out to a large public event, restaurant, shopping center, and socializing outside of the household are associated with lower mask use. In addition, stronger face mask-related policies are associated with higher mask usage. Taken together with existing evidence regarding the effectiveness of mask usage, our findings have important implications for health prevention policies and messaging in the context of the ongoing and future public health emergencies, as they highlight the importance of better targeting specific populations and behaviors when designing policies and messaging campaigns.

## Supplementary Information

**Additional file 1.**

## Data Availability

The survey data that support the findings of this study are available and publicly available on the University of Maryland’s website (https://covidmap.umd.edu/api.html). This website also has the survey questionnaire and detailed documentation of the data that are aggregated and uploaded daily. Individual-level data, which includes daily files with the survey variables and weights, are available for academic and nonprofit researchers upon request to the University of Maryland and Facebook. For instructions on how to submit an access form, please visit the website at https://covidmap.umd.edu/. Other data used in this study, such as those from Johns Hopkins University’s COVID-19 data repository and University of Oxford’s COVID-19 dataset are also publicly available online at https://github.com/CSSEGISandData/COVID-19, and https://www.bsg.ox.ac.uk/research/research-projects/coronavirus-government-response-tracker, respectively.
