# Supplementary material for: Global trends and predictors of face mask usage during the COVID-19 pandemic
Source: BMC Public Health. 2021 Nov 15;21:2099. doi: 10.1186/s12889-021-12175-9 (PMC8667772; doi:10.1186/s12889-021-12175-9)
Supplement: Supplementary file 1 — Additional file 1. [file 12889_2021_12175_MOESM1_ESM.docx]

**Global Trends and Predictors of Face Mask Usage During the COVID-19 Pandemic**

Elena Badillo-Goicoechea, MS^1^*, Ting-Hsuan Chang, BS^2^*, Esther Kim, PhD^3^, Sarah LaRocca, PhD^3^, Katherine Morris, PhD^3^, Xiaoyi Deng, MS^4^, Samantha Chiu, MA^4^, Adrianne Bradford, MS^4^, Andres Garcia, BS^4^, Christoph Kern, PhD^5^, Curtiss Cobb, PhD^3^, Frauke Kreuter, PhD^4,6,7^, Elizabeth A. Stuart, PhD^1,2^

^1^ Department of Mental Health, Johns Hopkins Bloomberg School of Public Health, Maryland, USA

^2^ Department of Biostatistics, Johns Hopkins Bloomberg School of Public Health, Maryland, USA

^3^ Facebook Research, California, USA

^4^ Joint Program in Survey Methodology, University of Maryland College Park, Maryland, USA

^5^ University of Mannheim, Germany

^6^ Department of Statistics, Ludwig Maximilians University, Munich, Germany

^7^ Institute for Employment Research, Nuremberg, Germany

* Contributed equally as first authors.

**Running title**: Global trends and predictors of face mask usage

**Word count**: Abstract (257), main manuscript excluding figures, tables, and acknowledgements (3643)

**Keywords**: COVID-19, SARS-CoV-2, face mask, mask usage

**Corresponding author**:

Elizabeth A. Stuart, PhD

**Address**:

Johns Hopkins Bloomberg School of Public Health

615 N. Wolfe St. W1033

Baltimore, MD 21205

**Email**:

[estuart@jhu.edu](mailto:estuart@jhu.edu)

**Phone**: 410-955-9088

**SUPPLEMENTARY MATERIALS**

**Supplementary Table 1**: List of countries, territories, and languages supported by the COVID-19 Symptom Survey

| **Countries or territories** | **Languages** |
| --- | --- |
| Andorra; United Arab Emirates; Afghanistan; Antigua and Barbuda; Anguilla; Albania; Armenia; Netherland Antilles; Angola; Antarctica; Argentina; American Samoa; Austria; Australia; Aruba; Azerbaijan; Bosnia and Herzegovina; Barbados; Bangladesh; Belgium; Burkina Faso; Bulgaria; Bahrain; Burundi; Benin; Saint Barthélemy; Bermuda; Brunei Darussalam; Bolivia (Plurinational State of); Bonaire, Sint Eustatius and Saba; Brazil; Bahamas; Bhutan; Botswana; Belarus; Belize; Canada; Congo, Democratic Republic of the; Central African Republic; Congo; Switzerland; Côte d'Ivoire; Cook Islands; Chile; Cameroon; China; Colombia; Costa Rica; Cuba; Cabo Verde; Curaçao; Cyprus; Czechia; Germany; Djibouti; Denmark; Dominica; Dominican Republic; Algeria; Ecuador; Estonia; Egypt; Western Sahara; Eritrea; Spain; Ethiopia; Finland; Fiji; Falkland Islands (Malvinas); Micronesia (Federated States of); Faroe Islands; France; Gabon; United Kingdom of Great Britain and Northern Ireland; Grenada; Georgia; French Guiana; Guernsey; Ghana; Gibraltar; Greenland; Gambia; Guinea; Guadeloupe; Equatorial Guinea; Greece; Guatemala; Guam; Guinea-Bissau; Guyana; Hong Kong; Honduras; Croatia; Haiti; Hungary; Indonesia; Ireland; Israel; Isle of Man; India; Iraq; Iran (Islamic Republic of); Iceland; Italy; Jersey; Jamaica; Jordan; Japan; Kenya; Kyrgyzstan; Cambodia; Kiribati; Comoros; Saint Kitts and Nevis; Korea, Republic of; Kuwait; Cayman Islands; Kazakhstan; Lao People's Democratic Republic; Lebanon; Saint Lucia; Liechtenstein; Sri Lanka; Liberia; Lesotho; Lithuania; Luxembourg; Latvia; Libya; Morocco; Monaco; Moldova, Republic of; Montenegro; Saint Martin (French part); Madagascar; Marshall Islands; Macedonia, the former Yugoslav Republic of; Mali; Myanmar; Mongolia; Macao; Northern Mariana Islands; Martinique; Mauritania; Montserrat; Malta; Mauritius; Maldives; Malawi; Mexico; Malaysia; Mozambique; Namibia; New Caledonia; Niger; Norfolk Island; Nigeria; Nicaragua; Netherlands; Norway; Nepal; Nauru; Niue; New Zealand; Oman; Panama; Peru; French Polynesia; Papua New Guinea; Philippines; Pakistan; Poland; Saint Pierre and Miquelon; Puerto Rico; Palestine, State of; Portugal; Palau; Paraguay; Qatar; Réunion; Romania; Serbia; Russian Federation; Rwanda; Saudi Arabia; Solomon Islands; Seychelles; Sudan; Sweden; Singapore; Saint Helena, Ascension and Tristan da Cunha; Slovenia; Svalbard and Jan Mayen; Slovakia; Sierra Leone; San Marino; Senegal; Somalia; Suriname; South Sudan; Sao Tome and Principe; El Salvador; Sint Maarten (Dutch part); Syrian Arab Republic; Eswatini; Turks and Caicos Islands; Chad; Togo; Thailand; Tajikistan; Tokelau; Timor-Leste; Turkmenistan; Tunisia; Tonga; Turkey; Trinidad and Tobago; Tuvalu; Taiwan, Province of China; Tanzania, United Republic of; Ukraine; Uganda; United States of America; Uruguay; Uzbekistan; Holy See; Saint Vincent and the Grenadines; Venezuela (Bolivarian Republic of); Virgin Islands (British); Virgin Islands (U.S.); Viet Nam; Vanuatu; Wallis and Futuna; Samoa; Kosovo; Yemen; Mayotte; South Africa; Zambia; Zimbabwe | Arabic; Azerbaijani; Bulgarian; Bengali; Czech; Cebuano; Danish; German; Greek; English (UK); NA; Spanish (Spain); Spanish; Persian; Finnish; French (Canada); French (France); Gujarati; Hebrew; Hindi; Croatian; Hungarian; Indonesian; Italian; Japanese; Kannada; Korean; Macedonian; Malayalam; Marathi; Malay; Burmese; Norwegian (bokmal); Dutch; Punjabi; Polish; Portuguese (Brazil); Portuguese (Portugal); Romanian; Russian; Slovak; Slovenian; Albanian; Serbian; Swedish; Swahili; Tamil; Telugu; Thai; Filipino; Turkish; Urdu; Vietnamese; Simplified Chinese (China); Traditional Chinese (Hong Kong); Traditional Chinese (Taiwan) |

**Supplementary Table 2**: Weighted distribution of respondent characteristics among 18,730,575 respondents who responded to the survey

|  | Overall  (Unweighted *N* = 18,730,575) | | | Mask usage = 1  (Unweighted *N* = 13,006,455) | | | Mask usage = 0  (Unweighted *N* = 3,655,440) | | |
| --- | --- | --- | --- | --- | --- | --- | --- | --- | --- |
| Sex | % | | | % | | | % | | |
| Female | 36.37 | | | 39.57 | | | 35.50 | | |
| Male | 45.83 | | | 49.61 | | | 55.12 | | |
| Other | 0.18 | | | 0.18 | | | 0.30 | | |
| Missing | 17.61 | | | 10.64 | | | 9.09 | | |
| Age | % | | | % | | | % | | |
| 18 – 34 | 13.01 | | | 13.45 | | | 13.63 | | |
| 25 – 34 | 21.77 | | | 23.98 | | | 20.78 | | |
| 35 – 44 | 15.07 | | | 16.77 | | | 16.64 | | |
| 45 – 54 | 14.66 | | | 16.35 | | | 16.92 | | |
| 55 – 64 | 8.93 | | | 9.59 | | | 11.32 | | |
| >= 65 | 9.97 | | | 10.33 | | | 12.84 | | |
| Missing | 16.59 | | | 9.52 | | | 7.88 | | |
| Current location | % | | | % | | | % | | |
| Urban | 43.26 | | | 48.99 | | | 38.97 | | |
| Non-urban | 38.36 | | | 39.45 | | | 51.34 | | |
| Missing | 18.38 | | | 11.56 | | | 9.69 | | |
| Gone to work outside in the last 24 hours | % | | | % | | | % | | |
| Yes | 32.33 | | | 35.40 | | | 39.31 | | |
| No | 61.42 | | | 62.61 | | | 58.73 | | |
| Missing | 6.25 | | | 1.99 | | | 1.95 | | |
| Gone to a market, grocery store, or pharmacy in the last 24 hours | % | | | % | | | % | | |
| Yes | 58.33 | | | 64.38 | | | 68.01 | | |
| No | 35.96 | | | 34.25 | | | 30.66 | | |
| Missing | 5.71 | | | 1.37 | | | 1.33 | | |
| Gone to a restaurant, café, or shopping center in the last 24 hours | % | | | % | | | % | | |
| Yes | 22.46 | | | 23.79 | | | 33.17 | | |
| No | 70.73 | | | 73.64 | | | 64.39 | | |
| Missing | 6.82 | | | 2.57 | | | 2.45 | | |
| Spent time with a non-same household member in the last 24 hours | % | | | % | | | % | | |
| Yes | 37.88 | | | 39.68 | | | 54.89 | | |
| No | 55.91 | | | 58.38 | | | 43.44 | | |
| Missing | 6.21 | | | 1.94 | | | 1.67 | | |
| Attended a public event with more than 10 people in the last 24 hours | % | | | % | | | % | | |
| Yes | 10.31 | | | 10.06 | | | 18.63 | | |
| No | 83.18 | | | 87.69 | | | 79.33 | | |
| Missing | 6.51 | | | 2.26 | | | 2.04 | | |
| Tested for COVID-19 | % | | | % | | | % | | |
| Yes | 13.72 | | | 14.05 | | | 11.31 | | |
| No | 83.50 | | | 85.03 | | | 87.82 | | |
| Missing | 2.77 | | | 0.93 | | | 0.88 | | |
| Worried about household finances in the next month* | % | | | % | | | % | | |
| Yes | 18.64 | | | 20.72 | | | 17.50 | | |
| No | 68.85 | | | 75.16 | | | 78.57 | | |
| Missing | 12.52 | | | 4.13 | | | 3.93 | | |
| Worked for pay in the last 7 days* | % | | | % | | | % | | |
| Yes | 44.49 | | | 49.46 | | | 54.35 | | |
| No | 41.95 | | | 45.25 | | | 40.70 | | |
| Missing | 13.56 | | | 5.29 | | | 4.94 | | |
| Years of education* | Q1 | Q2 | Q3 | Q1 | Q2 | Q3 | Q1 | Q2 | Q3 |
|  | 10 | 14 | 17 | 11 | 14 | 17 | 9 | 13 | 16 |

* Variable only included in the secondary model, fit with the narrower time period in which it was available.
